# Optimized method for the extraction of contaminant-free IgY antibodies from egg yolk using PEG 6000

**DOI:** 10.1016/j.mex.2022.101874

**Published:** 2022-10-06

**Authors:** Andrea Michelle Madera-Contreras, Roberto Solano-Texta, Alondra Cisneros-Sarabia, Isabel Bautista-Santos, Guillermina Vences-Velázquez, Amalia Vences-Velázquez, Karen Cortés-Sarabia

**Affiliations:** Facultad de Ciencias Químico-Biológicas of the Universidad Autónoma de Guerrero, Mexico

**Keywords:** IgY, Antibody, Hen, Extraction, PEG 6000

## Abstract

Hens are oviparous vertebrates and produce IgY antibodies, which is the main type of immunoglobulin in the egg yolk, and high concentrations can be obtained by using a simple method that does not require sophisticated equipment and reagents. The Polyethylene Glycol 6000 method allows the removal of lipids and the precipitation of IgY in two days with an approximated purity of around 80%, however during the original protocol other contaminant proteins can be precipitated. To overcome the issue of contamination with other proteins and extraction time, we optimized the previously method described by Pauly et al. (2011) by adding some changes that improved the aforementioned problems.

• Our protocol is customized by the addition of one more filtration step or one more step with PEG 6000 at 3.5% to avoid the contamination with lipids.

• Additionally, the changes in the type of agitation, centrifugation and the skip of dialysis make the method more accessible for all the laboratories.

• In summary, these modifications serve to enhance the purity, reduce the time for IgY extraction from egg yolk and make it more accessible for every basic research laboratory.

## Graphical abstract

**Figure:**
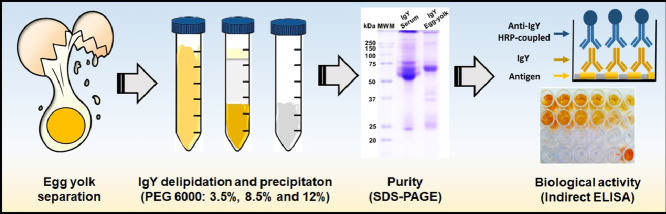

Specifications Table

**Table:** 

Subject Area	Immunology and Microbiology
More specific subject area	*Antibody purification*
Method name	Optimized method for IgY antibody purification using PEG 6000
Name and reference of original method	*IgY-extraction by means of PEG-precipitation* Our method is an optimized version of the proposed method by Diana Pauly in their publication [IgY Technology: Extraction of Chicken Antibodies from Egg Yolk by Polyethylene Glycol (PEG) Precipitation. Pauly, D., Chacana, P.A., Calzado, E.G., Brembs, B., Schade, R. J. Vis. Exp. 2011. 51: 1–6]. This protocol was first described by Polson et al. 1980 in their publication [Polson, A., von Wechmar, M.B and van Regenmortel, M.H. Isolation of viral IgY antibodies from yolks of immunized hens. Immunol. Commun. 1980. 9: 475–493]. All methods are based on the use of Polyethylene Glycol 6000 to remove lipids and precipitate IgY from the egg yolk.
Resource availability	*n/a*

## Background

The most used model for the production of IgY antibodies are hens. The molecular weight of IgY is around 180 kDa and is composed by two heavy (67 kDa) and two light (25 kDa) chains and its concentration in the egg yolk fluctuate from 5 to 15 mg/mL. Polyclonal antibody production in hens present relevant advantages as; phylogenetic distance between birds and mammals, no cross-reactivity with rheumatoid factors and complement proteins, economic (around 300 eggs per year), and the non-invasive obtainment method from the egg yolk [1], [2], [3].

**Fig. 1:**
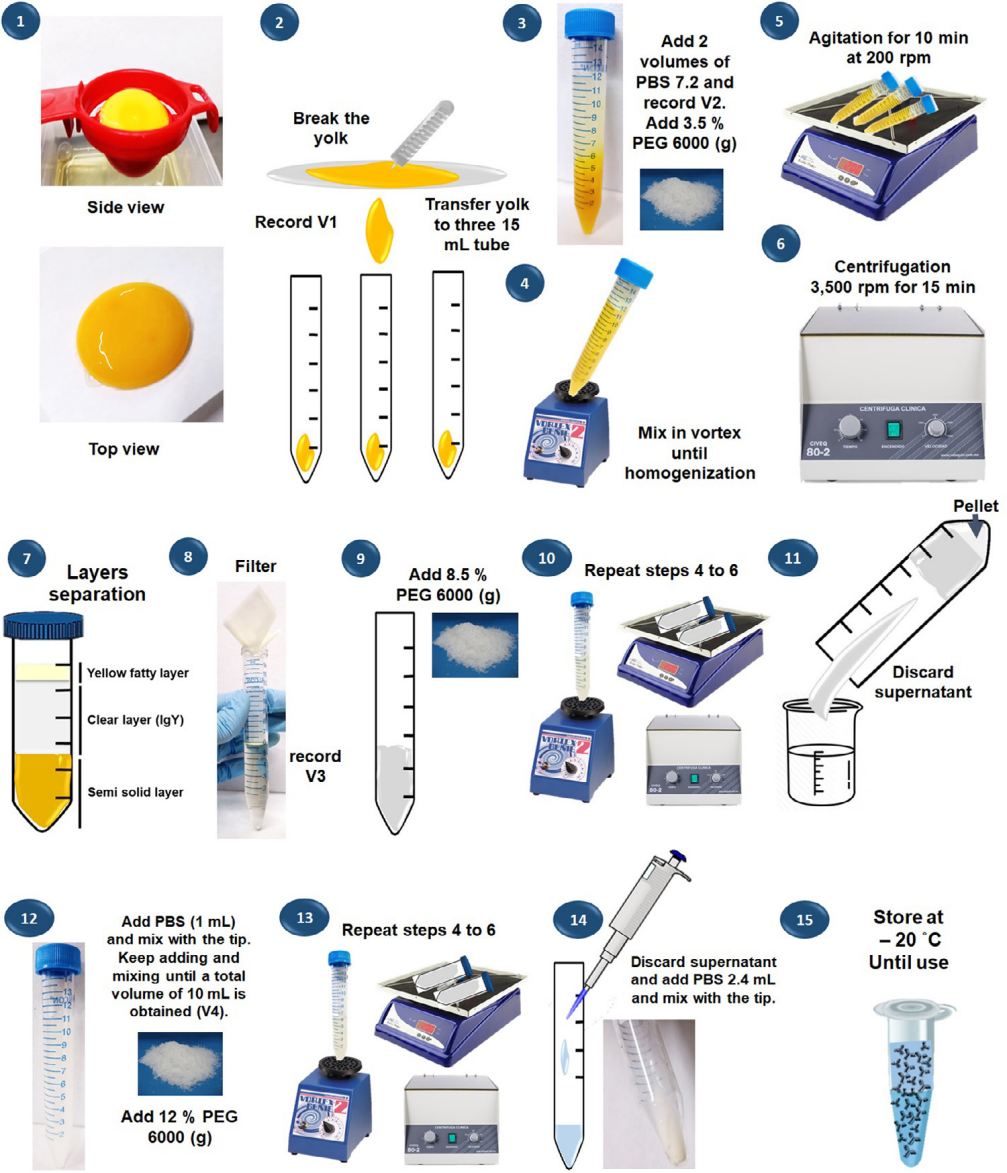
Schematic representation of the optimized method for IgY extraction from egg yolk using PEG 6000. The method was adapted from the previously described by Pauly et al. [6].

IgY are widely used in human and veterinary health as a therapy for viral, parasitic and fungal infections, also, their antiallergic, antitumoral and anti-venom activity has been tested. Whereas in the diagnostic field, the IgY has been used for the detection of specific antigens from microorganisms and tumoral cells [1]. The extraction of IgY from the egg yolk could be performed using water dilution method, polyethylene glycol (PEG) precipitation, caprylic acid, chloroform extraction, phenol extraction and carrageenan extraction [4]. The PEG method was first described by Polson et al., [5] and modified by Pauly et al., [6] and involves two different steps for the removal of lipids and the precipitation of total IgY, this method allows the obtainment of antibodies with high purity (around 60–80%), however some contaminant proteins can be observed. To overcome the problem of protein contamination and extraction time, we optimized the protocol by adding some steps listed in the Fig. 1 and adapted to be performed in any laboratory with the basic equipment.

## Required reagents and equipment

- Polyethylene Glycol 6000 (Sigma Aldrich Cat# 81255)
- Egg
- Sterile Phosphate buffered saline (PBS) pH 7.2
- Glass funnel
- Filter paper
- Yolk spoon
- Eppendorf tubes 1.5 mL
- Clinic centrifuge
- Nitrile gloves
- Falcon conical tubes (15 mL)

## Procedure

A full diagram of all the procedure for IgY extraction from the egg yolk is provided in the Fig. 1. This protocol is the optimized protocol of Pauly et al. [6].

(1) Break the eggshell very carefully and transfer the egg white and egg yolk to the yolk spoon and discard the egg white. Cut a piece of filter paper (15 × 15 cm) and then, place the yolk in the filter paper avoiding it from breaking. After, roll the yolk over the filter paper to remove remaining egg white.

**Note:** Use nitrile or latex gloves in all the procedure.

(2) Cut the skin on the yolk with a lancet and pour the liquid into three 15 mL tubes and measure the volume of each tube (which is considerate as volume 1 or V1).

**Note:** The egg yolk could be aspirated with a 10 mL syringe.

(3) Add twice the egg yolk volume (V1) of PBS and mix until a homogenous solution is formed (V2). Considering the V2, weight 3.5% of PEG 6000 (w/v) in grams and add to each 15 mL tube.
(4) Mix the solution during 2 min in the vortex.
(5) Mix the solution during 10 min in an orbital shaker at 200 rpm.
(6) Centrifuge at 3500 rpm for 15 min.
(7) After centrifugation, you will observe three layers and using a pipette take the two superficial layers (yellow fatty layer and clear layer that contains IgY).
(8) Pour the superficial layers twice through a folded filter paper and collect the filtrated supernatant into a new tube (V3).

**Note:** If remaining yellow fatty layer is still observed after second filtration, repeat the filter step one more time or repeat the 3.5% of PEG 6000 step.

(9) Considering the V3, weight 8.5% of PEG 6000 (w/v) in grams and add to the tube with the filtrated supernatant.
(10) Follow the same procedure as described in steps 4 to 6.
(11) Discard the supernatant.
(12) Add 1 mL of PBS using the tip to homogenize. Keep adding and mixing with the tip until a final volume of 10 mL is reached (V4). Considering the V4, weight 12% of PEG 6000 (w/v) in grams and add to the tube with PBS.
(13) Follow the same procedure as described in steps 4 to 6.
(14) Discard the supernatant and add 2.4 mL of PBS to dissolve the pellet and homogenize with the tip avoiding the formation of bubbles.

**Note:** If you want to increase the IgY concentration repeat the step 9 considering the volume of the supernatant as V4 and weight 12% PEG 6000.

(15) Divide the 2.4 mL into small aliquots (around 50 µL) and store at -20 °C until use. IgY are stable for around 1 year.

**Note:** Do not freeze at -70 °C and use one aliquot to quantify total proteins (mg/mL) by the election method.

## Method validation

**Fig. 2:**
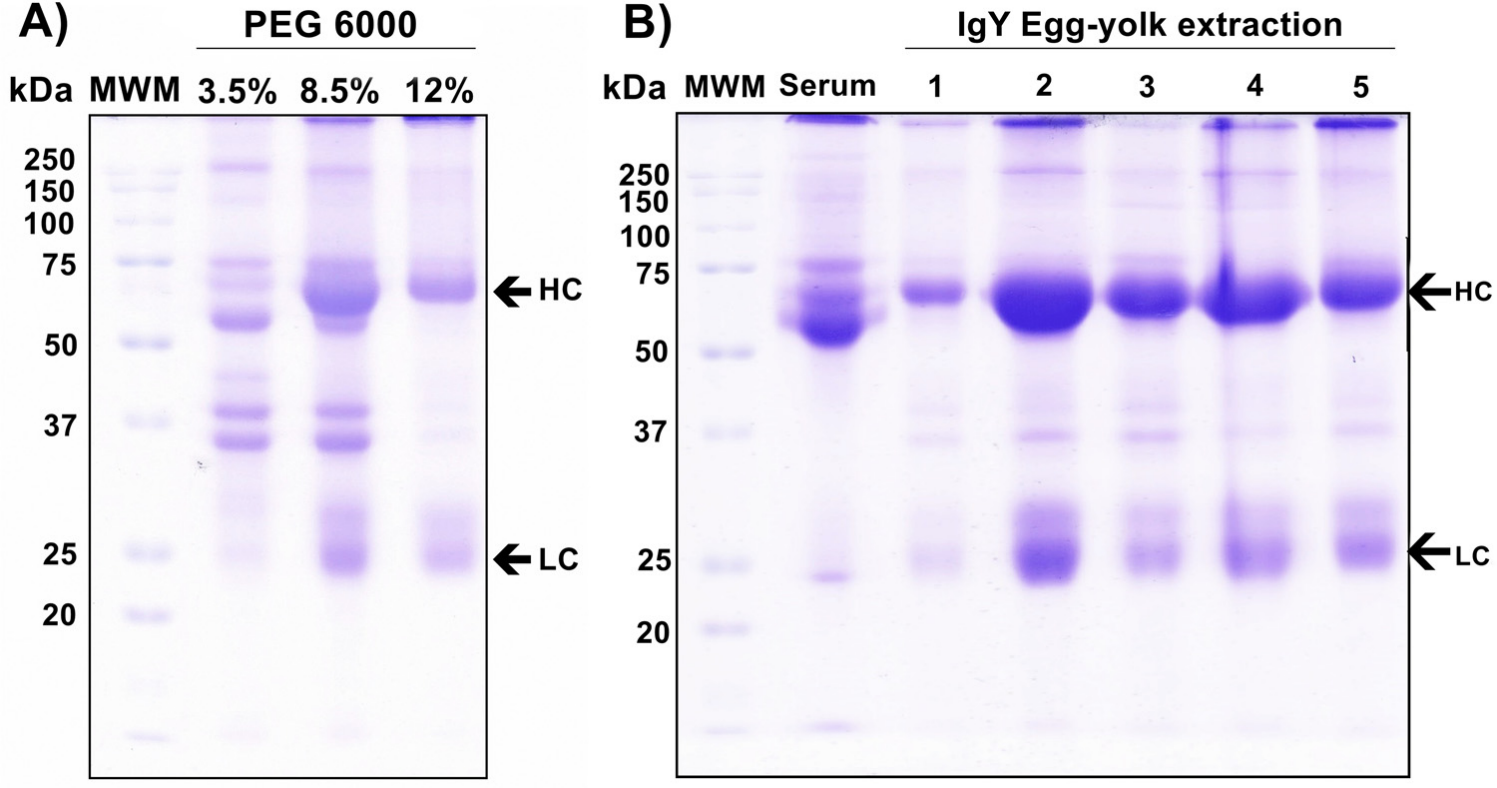
Evaluation of the purity of the extracted IgY from the egg yolk. A) Protein pattern of each one of the phases in which the PEG 6000 was used (3.5%, 8.5% and 12%). B) Reproducibility of the optimized method for IgY extraction. Lane 1: MWM (Molecular weight marker; All blue Biorad Cat##1610373EDU), lane 2: Serum sample, lanes 3–7: IgY samples derived from egg yolk using optimizing method. HC: heavy chain (around 65-70 kDa), LC: light chain (25 kDa).

To evaluate the final results of the optimized method for IgY extraction from the egg yolk using PEG 6000. First, we performed 12% SDS-PAGE and Coomassie blue staining using 5 µL of each one of the phases in which the PEG 6000 was used (3.5%, 8.5% and 12%). During the phases with 3.5% and 8.5%, multiple bands were observed, however, after the precipitation step (12%) the predominant bands have a molecular weight of around 70 kDa and 25 kDa that correlate with the heavy (HC) and light (LC) chains of IgY **(**Fig. 2 **Panel A)**. Later, we performed four addicional IgY extractions (2–5) to provide avidence about the reproducibility of the optimized method and performed a 12% SDS-PAGE and Coomassie blue staining to analyze the protein pattern of each extraction. As reference, we used one serum sample followed by the five extracted sampled. In the serum sample, multiple band were observed, whereas in the IgY extracted from the egg-yolk, the most predominant bands correlated with the HC (around 65–70 kDa) and LC (25 kDa) of IgY **(**Fig. 2 **Panel B)**.

**Fig. 3:**
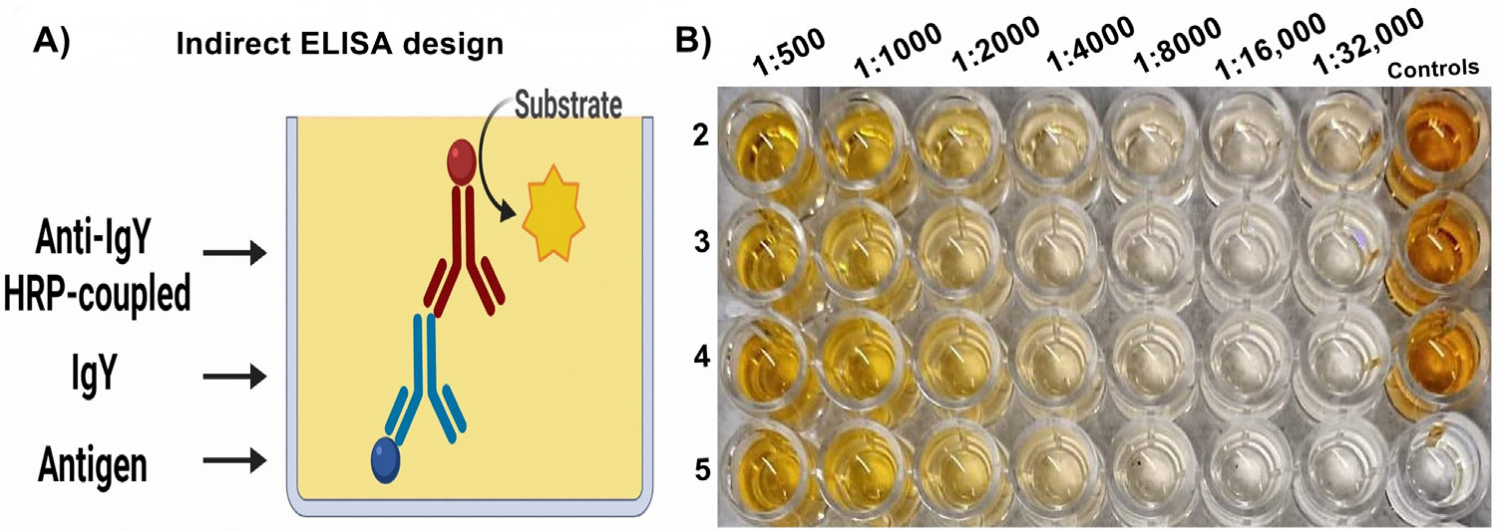
Biological activity of the extracted IgY using the optimized method. (A) Design of the Indirect ELISA used to test the biological activity of the extracted IgY. (B) ELISA results. Primary antibody: extracted IgY, secondary antibody: rabbit anti-IgY HRP-coupled (Sigma Aldrich #Cat A9046). In controls, we used IgY diluted (1:500) and omitted the primary antibody (white well) to test the background of the secondary antibody.

Also, we design an Enzyme-Linked ImmunoSorbent Assay (ELISA) to corroborate the biological activity of the extracted IgY. In this method, we fixed 100 µL of the antigen used for the immunization of the hen (Strain of ATCC 14018 of *Gardnerella vaginalis* 1 × 10^3^/mL at McFarland scale), after, we blocked the plate bottom with 5% skimmed milk diluted in PBS-Tween 20 0.05% for 30 min. Later, as primary antibody we used the four extracted IgY (showed in the lanes 4–7 of Fig. 3 **Panel B**) in serial dilutions from 1:500 to 1:32,000 and incubate the plate during 2 h at 37 °C. Finally, we used a rabbit anti-IgY HRP-coupled (Sigma Aldrich #Cat A9046) as secondary antibody diluted 1:4,000 in PBS-Tween 20 0.05% during two hours at 37 °C. Between each step, plate was washed three times with PBS-Tween 20 0.05%. Reaction was development with Orto-phenylenediamine (OPD: Sigma Aldrich Cat # P9029) and 30% hydrogen peroxide in citrate buffer (citric acid/sodium citrate 0.1 M) and stopped with 2N sulfuric acid and read at 492 nm **(**Fig. 3 **Panel A)**. Results corroborated that all the extracted IgY were able to recognize the antigen of interest at the same dilution (1:1,000), to which, we provided evidence about the biological activity of the extracted IgY **(**Fig. 3 **Panel B)**.

## Conclusions

The extraction of IgY from the egg yolk is an essential procedure for the obtainment of specific antibodies against the antigen. The PEG 6000 method allows the delipiding and precipitation of IgY. The current optimized protocol removes contaminant proteins and yield to the obtainment of samples with a purity >80%, also, the method does not require refrigerated centrifuge and the IgY could be extracted in around 4 h. Finally, the protocol is simple and can be performed with basic training without affecting the functionality of the antibody. We expect that this procedure will be of interest for investigators that perform continuous IgY extraction with research purposes (Table 1).

**Table 1:** Comparison of the optimized IgY method and that previously reported by Pauly et al. [6].

	Pauly et al. [6]	This study
**Steps**	**Delipidation procedure**	

Yolk separation	“yolk spoon”	“yolk spoon”
Mix	Vortex and 10 min on a rolling mixer.	Vortex and 10 min in an Orbital shaker (200 rpm).
Centrifugation	10,000 rpm for 20 min at 4 °C.	3500 rpm for 15 min at room temperature.
Phase separation	Supernatant poured through a folder filter and transferred to a new tube.	Supernatant poured through a folder filter twice and transferred to a new tube.
IgY precipitation	8.5% and 12% PEG 6000 (g), vortex and rolling for 10 min.	8.5% and 12% PEG 6000 (g), vortex and 10 min in an Orbital shaker (200 rpm).

	**IgY precipitation**	

Resuspension	0.8 mL PBS	2.4 mL PBS 7.2

	**Dialysis**	

Time	Overnight in 0.1% saline and three hours with PBS	No need
Volume	1.6 mL	
Container	Dialysis capsule	
	Pauly et al. [6]	This study

## Declaration of Competing Interest

The authors declare that they have no known competing financial interests or personal relationships that could have appeared to influence the work reported in this paper.

## Data Availability

Data will be made available on request.
